# *De novo KCNK4* variant caused epilepsy with febrile seizures plus, neurodevelopmental abnormalities, and hypertrichosis

**DOI:** 10.3389/fgene.2025.1499716

**Published:** 2025-03-31

**Authors:** Hong-Jun Yan, Wen-Hui Liu, Min-Xing Xu, Peng-Yu Wang, Yu-Jie Gu, Hua Li, Jing Guo, Sheng Luo

**Affiliations:** ^1^ Epilepsy Center, Guangdong Sanjiu Brain Hospital, Guangzhou, Guangdong, China; ^2^ Institute of Neuroscience, Key Laboratory of Neurogenetics and Channelopathies of Guangdong Province and the Ministry of Education of China, The Second Affiliated Hospital, Guangzhou Medical University, Guangzhou, Guangdong, China; ^3^ Department of Cardiology, Guangzhou Institute of Cardiovascular Disease, Guangdong Key Laboratory of Vascular Diseases, The Second Affiliated Hospital, Guangzhou Medical University, Guangzhou, China

**Keywords:** *KCNK4*, epilepsy with febrile seizures plus, phenotypic spectrum, spatiotemporal expression, phenotypic variation

## Abstract

**Background:**

Epilepsy with febrile seizures plus (EFS+) is a syndrome with a strong genetic component. Previously, variants in several genes encoding ion channels have been associated with EFS+. However, the etiology in the majority of patients remains undetermined.

**Methods:**

Trio-based whole-exome sequencing was performed on a patient with EFS+. Previously reported *KCNK4* variants were systemically reviewed to analyze the phenotypic spectrum and core phenotypes.

**Results:**

A novel *de novo KCNK4* variant (c.415G>A/p.Gly139Arg) was identified in a patient with EFS+, neurodevelopmental abnormalities, and hypertrichosis. The identified variant was absent in normal populations, indicated to alter hydrogen bonds with surrounding residues by various protein modeling, predicted to be damaging for protein function by twenty algorithms, located in residues of high conservation across species, and classified as pathogenic by the ACMG guidelines. Protein modeling analyses of the variant suggested a possible gain-of-function effect. Analysis of other eight cases with *KCNK4* variants outlined the phenotypic spectrums of *KCNK4*, ranging from mild benign epilepsy, EFS+ with neurodevelopmental abnormalities, to syndromic neurodevelopmental disorders and revealed neurodevelopmental abnormalities and epilepsy as its core phenotypes. Integrated analysis suggested that minor allele frequency and *in silico* meta-predictors effectively distinguish pathogenic variants.

**Conclusion:**

This study suggested the *KCNK4* gene as a novel candidate causative gene of EFS+, which would be helpful for the genetic diagnosis and clinical management of patients.

## Introduction

Epilepsy, a chronic neurological disorder characterized by recurrent seizures due to abnormal neuronal discharge in the brain, significantly impacts patients’ physical, psychological, and social wellbeing. It is estimated that approximately 70 million individuals worldwide are affected by epilepsy, making it one of the most prevalent neurological conditions (Trinka et al., 2019). Epilepsy encompasses a broad spectrum of seizure types and syndromes, each with distinct clinical manifestations, underlying mechanisms, and treatment responses (Katyayan and Diaz-Medina, 2021). The International League Against Epilepsy (ILAE) has classified epilepsy into various categories based on the seizure type, etiology, and age of onset, facilitating a more precise understanding of the disease and guiding therapeutic interventions (Scheffer et al., 2017).

Febrile seizures (FS) also known as febrile convulsions, represent a common and often transient neurological event that occurs in young children, particularly in those under the age of six (Mewasingh et al., 2020). These seizures are characterized by the sudden onset of convulsive movements, accompanied by loss of consciousness and muscle rigidity, and are triggered by a febrile illness, such as a viral or bacterial infection. (El-Radhi, 2024) FS are generally considered a benign outcome of immature brain development and resolve spontaneously with age, with the majority of children experiencing no long-term sequelae (Mewasingh et al., 2020). However, a subset of patients with FS may develop a more severe and persistent form of epilepsy known as epilepsy with febrile seizures plus (EFS+) (Khair and Elmagrabi, 2015). This syndrome is characterized by a familial predisposition, with a strong genetic component (Myers et al., 2018). The genetic underpinnings of EFS + have been extensively investigated in recent years, revealing complex functional pathways. Mutations in ion channel genes, particularly those encoding sodium, potassium, and chloride channels, have been implicated in the pathogenesis of this syndrome, such as *SCN1A*, *GABRA1*, *HCN1*, and *GABRG2* (Liu et al., 2023; Sun et al., 2008). However, these established genes could only explain a portion of EFS+ patients. Other causative genes conferring substantial risks on EFS+ remain to be discovered.

Recently, the *KCNK4* gene (MIM* 605720), encoding a member of the two-pore domain potassium (K2P) channel family, has garnered significant attention due to their crucial role in regulating neuronal excitability and synaptic transmission (Brohawn et al., 2012). Mutations in *KCNK4* have been implicated in a neurodevelopmental syndrome, namely facial dysmorphism, hypertrichosis, epilepsy, intellectual/developmental delay, and gingival overgrowth syndrome (FHEIG, MIM# 618381) (Bauer et al., 2018). To our knowledge, only four *KCNK4* variants have been reported to be associated with human diseases (Bauer et al., 2018; Garg et al., 2023; Mariani et al., 2021; Yan et al., 2022; Elhossini et al., 2024). The association between *KCNK4* and human diseases warrants further studies.

In this study, we identified a novel *de novo* pathogenic variant (c.415G>A/p.Gly139Arg) in the *KCNK4* gene in a pediatric patient with EFS+. The patient of this study presented partial features of the FHEIG syndrome, including neurodevelopmental abnormalities and hypertrichosis, but no facial dysmorphism and no gingival overgrowth. The pathogenicity of the identified *KCNK4* variant was analyzed by multiple methods, including various protein modeling, prediction made by twenty algorithms, cross-species sequence alignment, and classification of the guidelines of The American College of Medical Genetics and Genomics (ACMG). The underlying mechanism of phenotypic features was investigated by the spatiotemporal expression. Furthermore, the clinical features of previously published cases were systematically reviewed to analyze the underlying mechanism of phenotypic heterogeneity and outline the phenotypic spectrum of *KCNK4*.

## Material and methods

### Subjects

The patient was recruited from Guangdong Sanjiu Brain Hospital. Detailed clinical data were collected by face-to-face inquiries and physical examination, including developmental history, age of seizure onset, seizure manifestation, comorbidities, dysmorphic features, and response to antiseizure medicines, as well as electroencephalogram and brain magnetic resonance imaging (MRI) findings. Epileptic seizures and epilepsy syndromes were diagnosed based on the guidelines established by the International League Against Epilepsy’s Commission on Classification and Terminology (2022).

This study received approval from the ethics committee of the Guangdong Sanjiu Brain Hospital (AF/SW-07/01.0). Written informed consent was provided by the patient’s legal guardians.

### Whole exon sequencing

Blood specimens from the participant and his parents were procured. Whole-exome sequencing (WES) was performed utilizing the HiSeq 2000 system from Illumina (San Diego, CA, United States), adhering to rigorous sequencing protocols outlined in prior literature (Wang et al., 2022; Chen et al., 2022; Luo et al., 2023; Ye et al., 2024; Liu et al., 2022; He YL. et al., 2024; He et al., 2023; Jin et al., 2024; Jin et al., 2023). This process generated data through high-throughput parallel sequencing, achieving a mean coverage depth exceeding 100x and encompassing over 98% of the intended genomic regions. Subsequently, the raw sequencing outputs were aligned to the Genome Reference Consortium Human Genome Build 37 (GRCh37) reference sequence, facilitated by the Burrows-Wheeler alignment method. Upon successful alignment, single nucleotide polymorphisms (SNPs) and insertion/deletion variants (INDELs) were identified and annotated with the Genome Analysis Toolkit (GATK). The putative disease-causing variants in the family were pinpointed using a tailored analytical strategy (Luo et al., 2022; Tian et al., 2022a; Li et al., 2022; Tian et al., 2022b; Li et al., 2024). The *KCNK4* variant was annotated to the canonical transcript (NM_033310.2) and was validated by Sanger sequencing.

### Damaging effects analysis

The consequences of missense variants were predicted by multiple *in silico* tools with detailed scores acquired from the VarSome database (Kopanos et al., 2019). The conservation of mutated positions was evaluated through sequence alignment across species. Potential structural impacts of the variant were evaluated by conducting protein modeling using the three artificial intelligence tools, including the SWISS-MODEL software (April 2024 version, https://swissmodel.expasy.org/), the Phyre2 model (http://www.sbg.bio.ic.ac.uk/phyre2/html/page.cgi?id=index), and the AlphaFold model (monomer v2.0, https://alphafold.com/). The visualization and analysis of the protein’s three-dimensional structure were conducted using the PyMOL Molecular Graphics System (Version 2.3.2). To estimate the impact of protein stability of variants, the free energy change upon single nucleotide variants was analyzed via the three algorithms, including DynaMut, ENCoM, and SDM. The Δ vibrational entropy energy between wild-type and mutant was analyzed via DynaMut (https://biosig.lab.uq.edu.au/dynamut/prediction). The pathogenicity of variants was evaluated by the American College of Medical Genetics and Genomics guidelines (ACMG) (Richards et al., 2015).

### Assessment of spatiotemporal expression profile of *KCNK4*

Expression in 54 adult non-disease tissues was analyzed by the data from the GTEx database (https://www.gtexportal.org/home/). Human RNA-seq data of developmental stages (from 8 post-conceptional weeks to 40 years) for multiple brain areas were obtained from the Brainspan database (http://www.brainspan.org/). RNA expression was normalized to reads per kilobase million (RPKM). The expression spline is fitted by the locally weighted scatterplot smoothing (LOWESS) algorithm for interpreting the expression pattern of *KCNK4*.

### Assessing the pathogenicity of *KCNK4* variants using multiple indicators

*KCNK4* variants were systematically reviewed from the ClinVar database and classified into three categories: pathogenic/likely pathogenic (P/LP) variants, variants of uncertain significance (VUS), and benign/likely benign (B/LB) variants. To identify reliable indicators for assessing the pathogenicity of these variants, we compared the minor allele frequency (MAF) in gnomAD, the ΔΔG values from protein stability predictors, and the scores from the latest meta *in silico* predictors across the three variant groups.

### Statistical analysis

Statistical comparisons were conducted using appropriate statistical tests to assess the significance of differences in MAF, protein stability changes, and prediction scores between the different variant categories (P/LP, VUS, B/LB). Specifically, the Mann-Whitney U test was used for non-parametric data, while one-way ANOVA was employed for parametric data, followed by Tukey’s HSD test to determine which groups differed significantly. All these analyses were performed using the statistical software GraphPad Prism.

## Results

### Identification of *KCNK4* variant and clinical features of the patient

**FIGURE 1:**
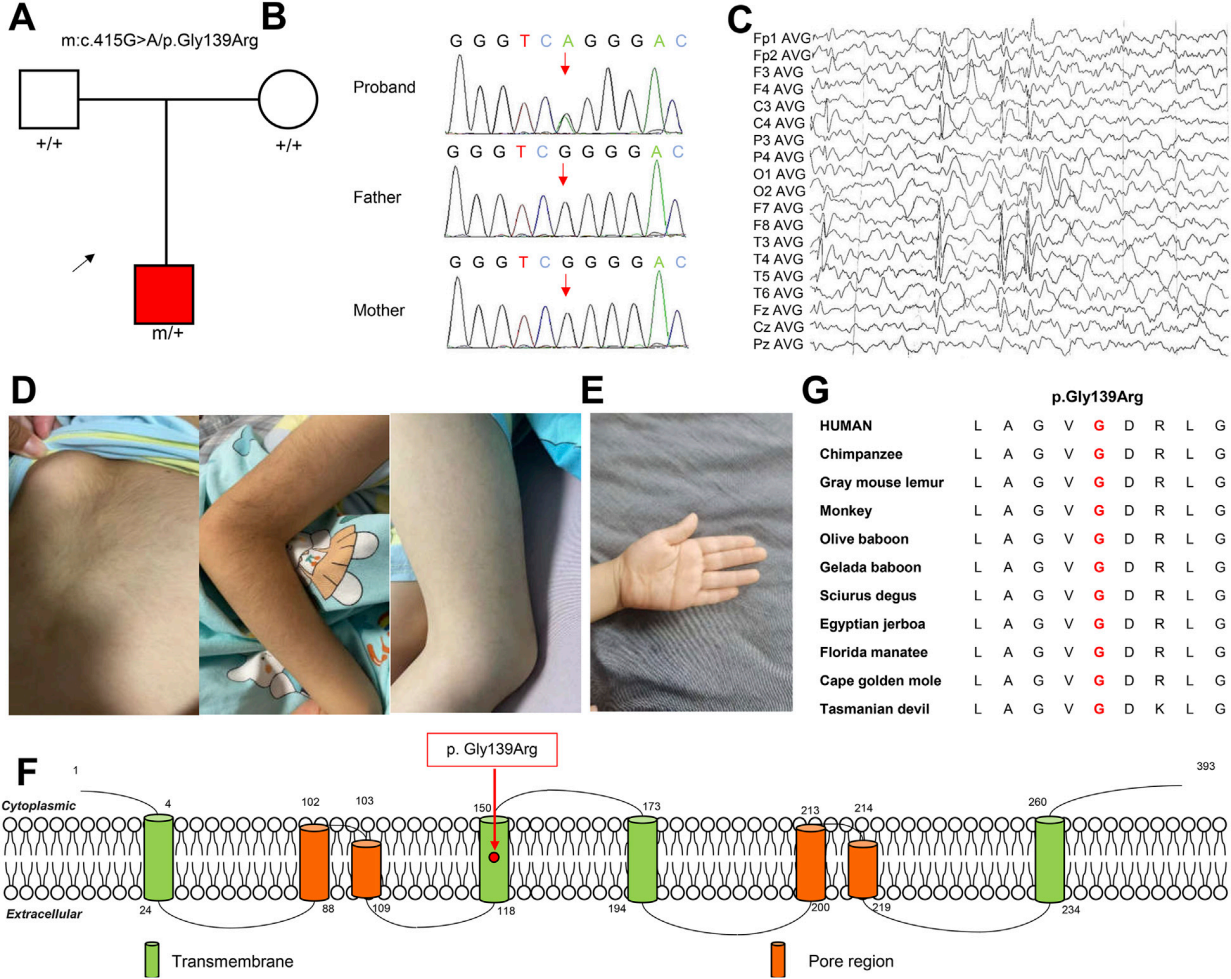
Genetic and clinical information of the identified *KCNK4* patient. **(A)** Pedigrees of the patient. **(B)** DNA sequence chromatograms of the identified *KCNK4* variant. **(C)** Representative EEG of the patient at the age of showed spike-slow waves in the Rolandic regions. **(D, E)** Clinical pictures of the patient showed hypertrichosis and simian crease. **(G)** Amino acid sequence alignment revealed that the missense variant was located in residues of high conservation across species. **(F)** Location of the identified variant in the KCNK4 channel.

In this study, one variant in the *KCNK4* gene, c.415G>A/p.Gly139Arg, was identified in a patient (Figures 1A, B). The variant was absent in the population’s database, including the gnomAD database, ExAC database Epi25 WES Browser, and the Han Chinese Genomes Database (Table 1). The identified *KCNK4* variant was not present in the patient’s parents either, confirming its *de novo* origin. Additionally, no “pathogenic”/“likely pathogenic” variants were identified in other established causative genes of epilepsy and neurodevelopmental disorders in this patient (Zhang et al., 2024).

**TABLE 1:** Minor allele frequency of the identified *KCNK4* variant c.415G>A/p.Gly139Arg.

Population	Allele count	Allele numbers	Allele frequency
gnomAD (V2.1.1)^a^	0	282,912	0
gnomAD (V3.1.2)^a^	0	152,312	0
gnomAD (V4.0.0)^a^	0	1,614,324	0
ExAC (V1.0)^a^	0	121,412	0
Epi25 WES Browser^b^	0	108,846	0
Han Chinese Genomes Database^c^	0	274,024	0

^a^ Data from the gnomAD database (https://gnomad.broadinstitute.org/).

^b^ Data from Epi25 WES Browser (https://epi25.broadinstitute.org/).

^c^ Data from Epi25 WES Browser (https://www.biosino.org/pgghan2/index).

The patient was a six-year-old male child, born to non-consanguineous parents via cesarean delivery. At 1.3 years of age, the child experienced a fever reaching 38.7°C after a lung infection, prompting the onset of his first seizure episode, characterized by focal to bilateral tonic-clonic seizures (FBTCS). Seizures were recurred eight times on that day, each lasting between 2 and 5 min. Thereafter, complex febrile seizures occurred annually, manifesting as multiple clusters of FBTCS, with each cluster comprising 2 to 10 seizures. Post-ictal, the patient displayed impaired responsiveness and unstable gait. At the age of 3, levetiracetam was taken orally and seizure-free status was achieved for 2 years. At the age of 5, simple febrile convulsions occurred again during sleep, the seizure was the same as before, but non-clustered. The last attack was at 5 years and 4 months old, with 4 focal afebrile seizures during sleep, showing the right side of the head and eyes, the right limb tetanic spasm, and the upper limb. Upon supplementation with lacosamide, seizure-free status was maintained for more than 4 months. EEG recording showed spike, spike-slow, and poly-spike-slow waves in bilateral central, partial, and posterior-temporal (Figure 1C). Brain MRI detected no gross structural abnormalities. He exhibited global developmental delays in early infancy. He also presented partial FHEIG features including hypertrichosis and facial dysmorphism manifesting deep eyehole and red and everted upper lip, but no obvious gingival overgrowth (Figure 1D). Additionally, this patient has a simian crease (Figure 1E).

### Evaluating the pathogenicity of the identified *KCNK4* variant

The identified variant, c.415G>A/p.Gly139Arg, was located in the second transmembrane region of the *KCNK4* protein (Figure 1F). Sequence alignment suggested that this variant affected a residue of high conservation across species (Figure 1G). Notably, the variant is predicted to be “damaging” or “conserved” by twenty *in silico* tools (Table 2). Additionally, the identified variant was located in the residue with a likely pathogenic variant that was reported in a previous study (p.Gly139Glu) (Yan et al., 2022). According to the ACMG guidelines, this variant could be classified as “pathogenic,” due to the origination of *de novo* (PS2), absent in normal controls (PM2), known pathogenic missense variants in this codon (PM5), and predicted to be damaging by multiple *in silico* tools (PP3).

**TABLE 2:** Pathogenic prediction of the identified *KCNK4* variant via 20 *in silico* algorithms.

Category		Algorithm	Score	Prediction	Explanation
Meta predictors		MetaRNN	0.9405	Pathogenic Strong	These predictors determine pathogenicity based on the combined evidence from multiple other *in silico* predictors
		BayesDel addAF	0.3027	Pathogenic Moderate	
		BayesDel noAF	0.197	Pathogenic Supporting	
		REVEL	0.755	Pathogenic Supporting	
Individual predictors		AlphaMissense	0.9731	Pathogenic Moderate	This is purely a simple representation of the scores to give a quick visual overview, the Germline Variant classifier uses a more sophisticated approach and doesn’t double-count
		EIGEN	0.9719	Pathogenic Moderate	
		EIGEN PC	0.8896	Pathogenic Moderate	
		Mutation assessor	3.86	Pathogenic Moderate	
		MutPred	0.784	Pathogenic Moderate	
		Fathmm-MKL	0.9815	Pathogenic Supporting	
		FATHMM-XF	0.8992	Pathogenic Supporting	
		LIST-S2	0.9794	Pathogenic Supporting	
		LRT	0	Pathogenic Supporting	
		PROVEAN	−6.34	Pathogenic Supporting	
		SIFT	0	Pathogenic Supporting	
		SIFT4G	0.001	Pathogenic Supporting	
		CADD	33	Pathogenic	
Conservation predictors		PhastCons100way	1.000	Conserved	
		PhyloP100way	7.335	Conserved	
		GERP	5.36	Conserved	

Note: Engines are assigned a prediction points score based on the strength of the calibrated prediction. Supporting: 1 point. Moderate: 2 points. Strong: 4 points. Very Strong: 8 points. Detailed score was obtained from the VarSome database.

### Exploring the possible pathogenic mechanism of the variant *via* protein modeling

**FIGURE 2:**
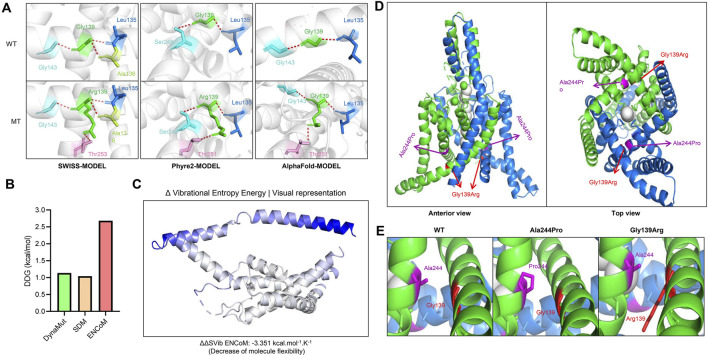
Molecular impact of the identified *KCNK4* variant. **(A)** Alteration of hydrogen bonds caused by the identified *KCNK4* variant in three artificial intelligence models. **(B)** Protein stability changes of the variant were analyzed by three algorithms. The DDG value greater than 0.5 kcal/mol means significantly increased protein stability. **(C)** Protein flexibility calculated by the DynaMut algorithm, which was indicated by the Δ vibrational entropy energy. The bluer the color of the structure is, the more rigid it is. **(D)** The molecular structure of the KCNK4 channel (PDB: 4WFE) and the location of the variants p.Ala244Pro and p.Gly139Arg. **(E)** The sterical obstacle between the second and the fourth transmembrane segments caused by the variants p.Ala244Pro and p.Gly139Arg.

The molecular effects of the missense variants were preliminarily analyzed by protein modeling through three advanced artificial intelligence algorithms: the SWISS-MODEL, the Phyre2, and the AlphaFold model. The missense variant was predicted to alter hydrogen bonds with surrounding residues calculated by all three models. (Figure 2A). Specifically, the variant p.Gly139Arg caused a newly formed hydrogen bond with residue Thr253 in the SWISS model, while a newly formed hydrogen bond with residue Thr251 was exhibited in the Phyre2 and AlphaFold models. Such a structural alteration would have an impact on the protein stability. As expected, the variant is predicted to cause an abnormally increased protein stabilization, analyzed by the DynaMut, ENCoM, and SDM algorithm (Figure 2B). Similarly, such a stabilizing change would decrease molecule flexibility, as indicated by the Δ vibrational entropy energy (ΔΔSVib ENCoM) of −3.351 kcal/mol/K (Figure 2C).

Previous studies have resolved the crystal structures of the *KCNK4* channel for both conducting and non-conducting states (Rietmeijer et al., 2021; Lolicato et al., 2014; Brohawn et al., 2014), revealing the gating mechanism of mechanical stimuli. To further elucidate the potential impact of the p.Gly139Arg variant on the KCNK4 channel function, it is crucial to understand the structural features and gating mechanisms of the Functional K2P4.1 channel (Liao et al., 2022). The KCNK4 channels are composed of four transmembrane segments (TM1–4) and two pore domains (P1–2). Among these components, the TM4 and the TM2-TM3 linker are particularly crucial for stabilizing the channel’s conductive conformation. In the conductive state, TM4 rotates around a central hinge, effectively sealing the intramembrane cavity to prevent lipid occlusion and enable ion permeation. This intricate mechanism maintains an unimpeded pathway for potassium ions, ensuring optimal channel function. The channel forms homodimers through domain swapping, allowing potassium ions to pass through lateral openings (Figure 2D).

In this study, the identified *KCNK4* variant p.Gly139Arg was located in the second transmembrane segment, replaced the glycine with a large, positively charged residue, decreased the flexibility of the second transmembrane segments, which may affect the conformation of the fourth transmembrane segments. Previously, a pathogenic variant in the fourth transmembrane helix, p.Ala244Pro, has been reported (Bauer et al., 2018). Whole-cell electrophysiological assay revealed that membrane currents recorded from cells transfected with the mutant channels p.Ala244Pro were substantially larger and more prominent within the negative potential range (Bauer et al., 2018). These findings indicated a d gain-of-function effect of p.Ala244Pro. Interestingly, the residue Ala244 is located closely with the residue Gly139 in the *KCNK4* crystal structures. The two variants introduced a similar sterical obstacle between the second and the fourth transmembrane segments, which may affect the conformational regulation of the fourth transmembrane segments (Figure 2E). The variant p.Gly139Arg may produce a functional impact of gain-of-function effects, similar to the variant p.Ala244Pro.

### Emerging phenotypic spectrum and core phenotypes revealed by reviewing previously reported *KCNK4* cases

**FIGURE 3:**
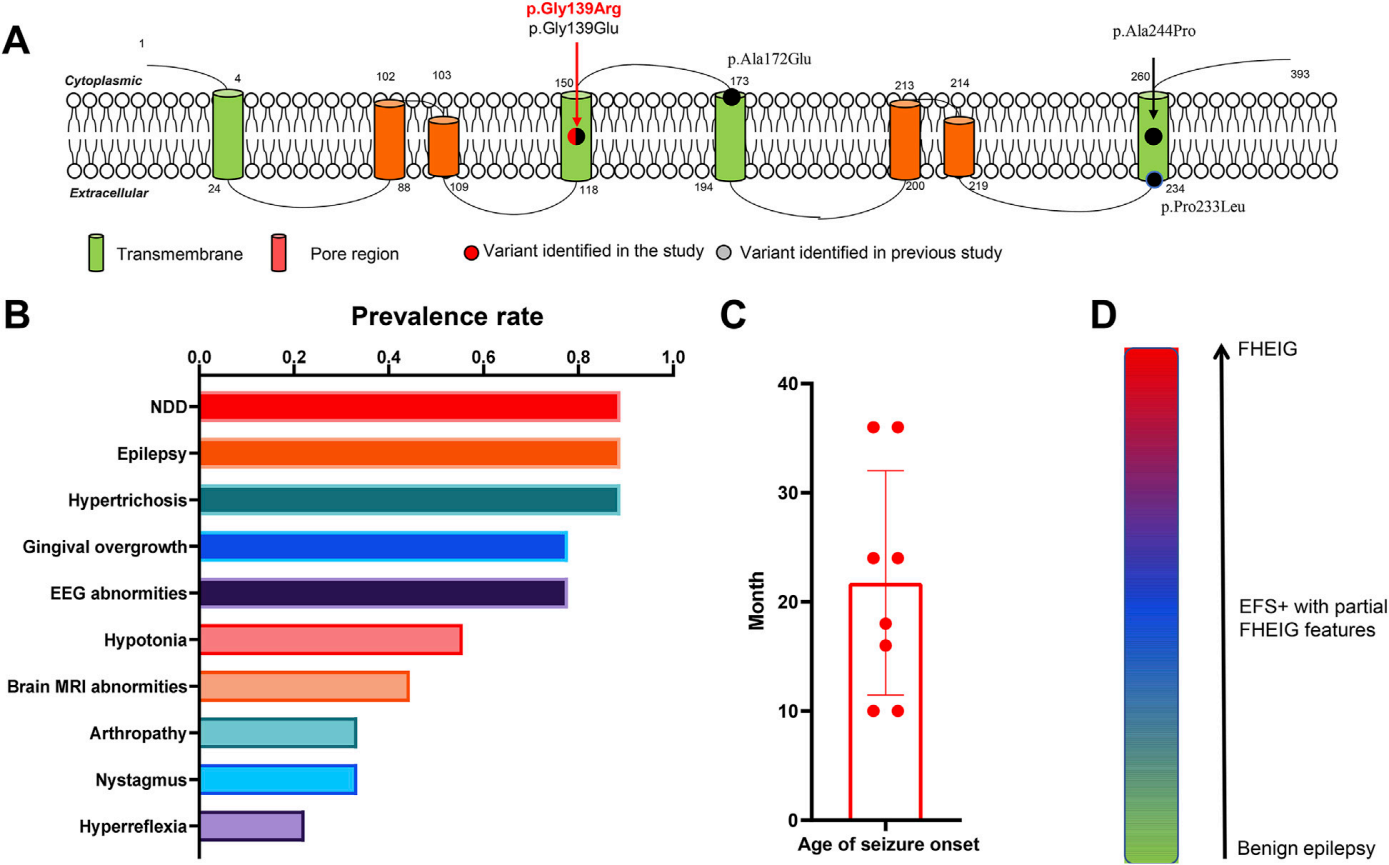
Emerging phenotypic spectrum and core phenotypes of *KCNK4*. **(A)** Location of all identified *KCNK4* variants. **(B)** Putative phenotypic spectrum of *KCNK4*. **(C)** The prevalence rate of phenotypes among patients with *KCNK4* variants. **(D)** The ages of seizures of patients with *KCNK4* variants.

So far, a total of four heterozygous pathogenic or likely pathogenic *KCNK4* variants were reported in eight unrelated cases (Figure 3A; Table 3, data obtained up to August 2024). Seven patients presented typical features of the FHEIG syndrome (Bauer et al., 2018; Garg et al., 2023; Mariani et al., 2021; Yan et al., 2022; Elhossini et al., 2024), while a patient exhibited Rolandic epilepsy (Yan et al., 2022). Among these patients, neurodevelopmental abnormalities, epilepsy, and hypertrichosis emerged as the higher prevalence phenotypes, as well as varied facial abnormalities (Figure 3B). Specifically, the neurodevelopmental phenotype consisted of intellectual impairment of varied severity, hypotonia, and varied motor and language development delays. Epilepsy was presented in 7 patients, with age of seizure onset ranging from 10 months to 3 years (Figure 3C). One patient was not reported to have seizures. This patient was evaluated at 11 months, an age potentially lower than the average age of seizure onset in other patients. Focal or focal-originated seizures were the most common seizure type, and two patients presented with also generalized seizures. Two patients exhibited refractory seizures, while the other five patients achieved seizure-free status through treatment with sodium channel blockers alone or in combination with valproate.

**TABLE 3:** Clinical features of patients with *KCNK4* variants.

Variants	c.415G>A, p. (Gly139Arg)	c.416G>A, p. (Gly139Glu)	c.515C>A, p. (Ala172Glu)			c.698C>T, p. (Pro233Leu)	c.730G>C, p. (Ala244Pro)		
Patient	1	2	3	4	5	6	7	8	9
Reference	This study	Yan et al. (2022)	Bauer et al. (2018)	Bauer et al. (2018)	Garg et al. (2023)	Yan et al. (2022)	Mariani et al. (2021)	Bauer et al. (2018)	Elhossini et al. (2024)
Inheritance	*de novo*	*de novo*	*de novo*	*de novo*	*de novo*	*de novo*	*de novo*	*de novo*	Inherited from the affected mother
Sex	Male	Male	Male	Female	Female	Male	Male	Male	Male
Age at last evaluation	6 years	5 years 4 months	11 months	8 years	Early adolescent	10 years	6 years	5 years 7 months	5 years
Neurological phenotypes									
Neurodevelopment	GDD	GDD, severe ID	Severe DD/ID	GDD/severe ID	Mild DD, learning difficulties, behavioral and emotional issues	Normal	Mild language delay	Low average, mild speech delay; fine motor delay	IQ score of 75 (borderline)
Age of seizure onset	1 year 4 months	3 years	NA	2 years	3 years	1.5 ears	10 months	10 months	2 years
Seizure type	FS, FBTCS	FBTCS	NA	GTCS, Absence	FBTCS, focal seizures	Focal seizures	Focal seizures	FBTCS	GTCS
Outcomes	Seizure-free on LEV and LCM	Seizure-free on VPA and OXC	NA	Seizure-free on LEV, OXC, CBZ, CLN, Pyridoxine	Refractory	Seizure-free on VPA and OXC	Seizure-free on CBZ	Seizure-free on CBZ	Refractory
EEG	Interictal: spike, spike-slow, and poly-spike-slow waves in Rolandic regions	Diffuse 4–5 Hz slow waves and spikes-slow waves in the right anterior head	Medium-voltage asymmetric theta activity over the right temporal areas. Low-voltage fast activity over the frontal regions followed by abundant high- voltage theta activity (frontal and centrotemporal areas) in sleep. Diffuse slow wave activity without epileptic discharges	ND	Semirhythmic spikes and slow waves; epileptiform spikes in the left hemisphere	Ictal: focal discharges in Rolandic regions	ND	Ictal: epileptic discharges from left parietal-temporal and occipital regions with rapid diffusion. Interictal: normal	Generalized epileptogenic activity
Brain MRI	Normal	Normal	Thin corpus callosum and ventricular enlargement	ND	Normal	Normal	Small-sized adenohypophyseal gland, otherwise normal	Mild enlargement of bilateral frontal-insular and temporal subarachnoid spaces	Wide cerebrospinal fluid space
Hypotonia	No	Yes	Yes	Yes	Yes	No	ND	No	Yes
Hyperreflexia	No	Yes	Yes	No	No	No	No	No	No
Other phenotypes									
Facial abnormalities	Yes	Yes	Yes	Yes	Yes	No	Yes	Yes	Yes
Gingival overgrowth	No	Yes	Yes	Yes	Yes	No	Yes	Yes	Yes
Hypertrichosis	Yes	Yes	Yes	Yes	Yes (mild)	No	Yes	Yes	Yes
Other features	Simian crease	No	No	Pierre Robin sequence cleft palate; brachydactyly; congenital hip dysplasia; intention tremor	Strabismus	No	No	No	Generalized joint laxity

Abbreviation: CBZ, carbamazepine; CLB, clobazam; CLN, clonazepam; DD, developmental delay; F, female; FBTCS, focal to bilateral tonic-clonic seizure; GDD, global developmental delay; GTCS, generalized tonic-clonic seizure; ID, intellectual disability; LEV, levetiracetam; LCM, lacosamide; LTG, lamotrigine; M, male; NA, not applicable; ND, no data; OXC, oxcarbazepine; STM, sulthiame; VPA, valproate.

The patient of this study presented partial features of the FHEIG syndrome, including neurodevelopmental abnormalities and hypertrichosis, but no facial dysmorphism and no gingival overgrowth. Additionally, the epileptic phenotypes in the case differed from those in previously reported cases, manifesting as typical EFS+ with a response to levetiracetam and lacosamide.

By reviewing these cases, we have identified an emerging phenotypic spectrum of *KCNK4* ranging from mild epilepsy, moderate EFS+ with partial FHEIG features, to severe FHEIG syndrome (Figure 3D). Neurodevelopmental abnormalities, epilepsy, and hypertrichosis would be its core phenotypes. However, there is also variability in the presentation of these phenotypes, as demonstrated by the differences presented in the current case and previously reported cases.

### Spatiotemporal expression of *KCNK4* and its association with phenotypic features

**FIGURE 4:**
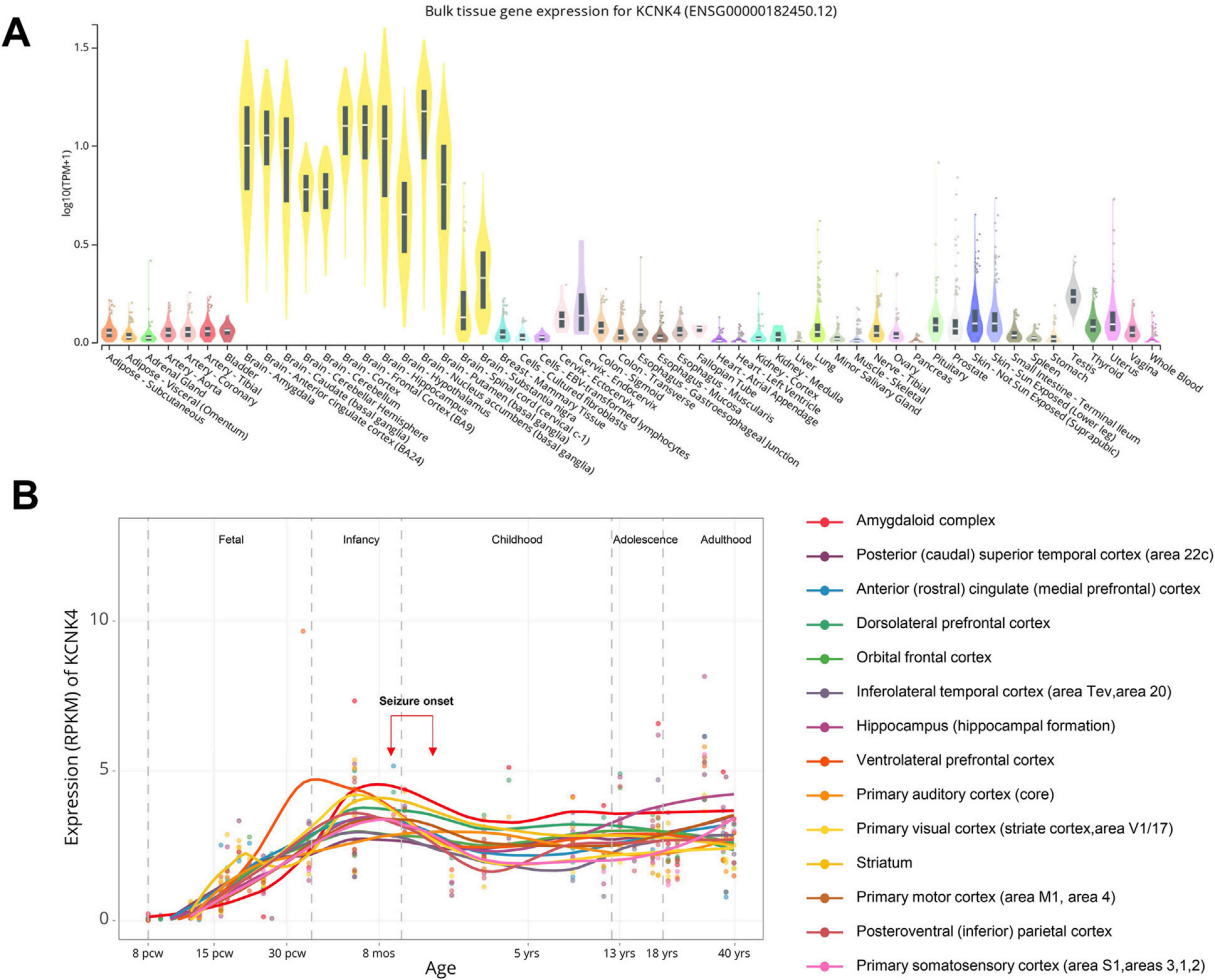
The spatiotemporal expression of *KCNK4*. **(A)** Bulk tissue gene expression for *KCNK4* (ENSG00000182450.12). Data were obtained from the GTEx database. **(B)** Expression profile of *KCNK4* across brain regions at different developmental stages. Data were obtained from the BrainSpan database Abbreviations: pcw, post-conception weeks; mos, months; y, years.

The expression, as one of the core characteristics of genes, is closely linked to the phenotypes they cause, especially epilepsy-associated genes (Chi and Kiskinis, 2024). We thus analyzed the spatio-temporal expression of *KCNK4*, to investigate the mechanism underlying phenotypic heterogeneity. The *KCNK4* gene is biasedly expressed in multiple brain tissues, including the brain cortex, frontal cortex, hippocampus, and other brain regions, with a very low level in other tissues, such as the skin. (Figure 4A, GTEx database). This biased expression pattern in the brain is potentially one of the explanations for neurodevelopmental abnormalities and epilepsy as the phenotypic core of the *KCNK4* gene.

Furthermore, recent studies have implicated the temporal expression, also known as the genetic dependent stage (GDS), in the onset ages and outcomes of diseases (He MF. et al., 2024; Li et al., 2023; Fan et al., 2024). We investigated the GDS of *KCNK4* across different brain regions. The *KCNK4* expression increased from the fetal stage, peaked in later infancy, slightly decreased in early childhood, and then maintained a stable and high expression throughout later stages (Figure 4B, Brainspan database). This pattern of high expression during later infancy and early childhood suggests a critical functional role for *KCNK4* during these periods. Clinically, patients with *KCNK4* variants exhibited a wide range of seizure onset ages, spanning from 10 months to 3 years, aligning with the peak expression of *KCNK4* in later infancy. Furthermore, the refractory seizures in three patients could potentially be explained by the stable and high expression of *KCNK4* maintained throughout later stages.

### Exploring the possible reliable indicator in assessing the pathogenicity of *KCNK4* variants

**FIGURE 5:**
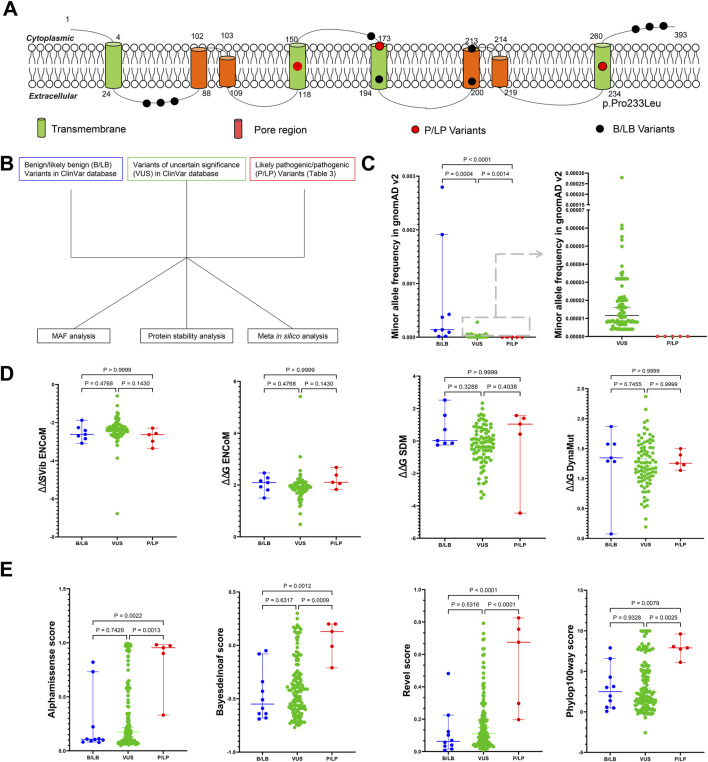
Exploring the possible reliable indicator in assessing the pathogenicity of *KCNK4* variants. **(A)** Location of all identified pathogenic and likely pathogenic (P/LP) variants and benign/likely benign *KCNK4* variants. **(B)** Flow chart of the pathogenic prediction of *KCNK4* variants. **(C)** Minor allele frequency comparison between P/LP variants, B/LB variants, and variants of uncertain significance (VUS). **(D)** Comparison of protein stability changes predicted by multiple algorithms between P/LP variants, B/LB variants, and VUS. **(E)** Comparison of *in silico* Meta-predicted scores between P/LP variants, B/LB variants, and VUS.

Accurately assessing the pathogenicity of genetic variants is crucial for the diagnosis and management of genetic disorders. To identify reliable indicators for assessing the pathogenicity of *KCNK4* variants, we analyzed the features of P/LP, VUS, and B/LB variants using multiple parameters (Figures 5A, B; Supplementary Table S1). MAF analysis revealed significant differences among these variant categories. Specifically, the identified P/LP variants were absent from population databases, exhibiting significantly lower MAF compared to both VUS (P = 0.0004) and B/LB variants (P < 0.0001) (Figure 5C). Furthermore, VUS displayed a significantly lower MAF compared to B/LB variants (P = 0.0014), suggesting natural selection pressures on these variants. In addition to MAF, we evaluated protein stability changes predicted by multiple algorithms, including DynaMut, ENCoM, SDM, and ΔΔSVib ENCoM. However, no significant differences were observed among the P/LP, VUS, and B/LB variants in terms of protein stability predictions (Figure 5D). To further discriminate among these variants, we employed the latest Meta *in silico* predictors, such as Alphamissense, Bayesdelnoaf, Revel, and Phylop100way. These predictors demonstrated excellent performance in distinguishing P/LP variants, although the difference between VUS and B/LB variants was not significant (Figure 5E). These findings underscore the potential utility of MAF and Meta *in silico* predictors as reliable indicators in assessing the pathogenicity of newly identified *KCNK4* variants. In summary, our comprehensive analysis suggests that integrating MAF data with predictions from advanced Meta *in silico* algorithms could enhance the accuracy of *KCNK4* variant classification. This approach would be helpful in facilitating more precise genetic counseling and clinical management for patients with suspected *KCNK4*-related disorders.

## Discussion

In this study, we identified a novel *de novo* variant p.Gly139Arg in the *KCNK4* gene. The pathogenicity of the identified *KCNK4* variant was supported by multiple pieces of evidence, including the affected residue of high conservation, hydrophobicity alteration, predicted to be “damaging” or “conserved” by 20 *in silico* tools, and classification of “pathogenic” variant by the ACMG guidelines. The protein modeling indicated that this variant caused significant alteration in hydron bonds, protein flexibility, and potential gain-of-function effects speculated from the stereochemical obstacle similar to the previously characterized pathogenic variant p.Ala244Pro. The patient exhibited typical EFS+ with partial features of FHEIG, including neurodevelopmental abnormalities and hypertrichosis, but no facial dysmorphism and gingival overgrowth. Reviewing previously reported cases revealed an emerging phenotypic spectrum of *KCNK4*. This study suggested that *KCNK4* is potentially a novel causative gene of EFS+, which would be helpful for the genetic diagnosis and clinical management of patients.

As a common epileptic syndrome in children, EFS+ is generally considered to be genetically determined, due to its high prevalence within family cases. Research into the genetic underpinnings of EFS + has identified multiple causative/susceptibility genes (evaluated by the OMIM database), among which the majority were ion channel genes, such as voltage-gated sodium channel genes (*SCN1A* and *SCN1B*), γ-aminobutyric acid receptor subunit genes (*GABRA1*, *GABRD*, and *GABRG2*), and hyperpolarization-activated cyclic nucleotide-gated potassium channel genes (*HCN1* and *HCN2*). Clinically, the etiology in many patients with EFS + remains elusive (Polizzi et al., 2012). This study identified a novel *de novo KCNK4* variant in a patient with EFS+. The pathogenicity of the identified *KCNK4* variant was supported by multiple pieces of evidence and could be classified as “pathogenic” by the ACMG guidelines. Furthermore, no “pathogenic”/“likely pathogenic” variants were identified in other established causative genes of epilepsy in this patient. The variant was thus considered to be the genetic cause for the patient, indicating that the *KCNK4* gene is potentially a novel causative gene of EFS+.

The majority of patients with EFS+ presented a favorable outcome, while a portion of patients presented with also refractory seizures (Camfield and Camfield, 2015). Due to the possibility of aggravating seizures, sodium channel blockers were typically prohibited in the clinical management of EFS+, particularly those caused by *SCN1A* variants. However, sodium channel blockers were proved to be effective in patients with EFS + but without *SCN1A* variants (Liu et al., 2023; Scheffer, 2024). Furthermore, sodium channel blockers present favorable responses to many potassium channel-related epilepsies, such as those caused by *KCNQ2* (Zimmern et al., 2022). Analysis of the previously reported cases indicated that sodium channel blockers solely or in combination with valproate were commonly used in the *KCNK4* patients, who achieved seizure-free status. It is possible that the application of sodium channel blockers would also be effective for patients with EFS + caused by *KCNK4* variants, which warrants clinical trials. Early genetic diagnosis would help the clinical management of patients with EFS+.

Previously, the *KCNK4* gene has been reported to be associated with the FHEIG syndrome, which is featured by neurological phenotypes of intellectual disability and epilepsy, and the non-neurological phenotypes of facial dysmorphism, hypertrichosis, and gingival overgrowth (Bauer et al., 2018). So far, four *KCNK4* variants have been reported in eight patients. Seven patients exhibited classic phenotypes of FHEIG syndrome, while a patient presented only epilepsy. In this study, the patient exhibited partial features of FHEIG syndrome, including intellectual disability, epilepsy, and hypertrichosis. The observed phenotypes in patients suggested the neurological phenotypes as the core features of *KCNK4*-related diseases, which could be understood from the biased expression in the brain and low expression in other tissues. The *KCNK4* gene is potentially associated with a spectrum of phenotypes, ranging from mild isolated epilepsy, moderate EFS+ with partial FHEIG features, to severe FHEIG syndrome, which needs further studies in large cohorts.

The integrated analysis conducted in this study underscores the significance of *in silico* prediction tools in assessing variant pathogenicity. By examining P/LP variants, VUS, and B/LB variants, we observed that the minor allele frequency and *in silico* Meta algorithm exhibited notable proficiency in distinguishing between pathogenic and non-pathogenic variants. This finding aligns with previous research indicating the reliability of these computational methods. However, it is important to acknowledge that *in silico* predictions are not infallible; they are based on existing data and algorithms, which may have limitations or biases. Therefore, while our results highlight the utility of these tools, they should be interpreted with caution and in conjunction with other evidence, such as clinical data and functional studies. Nonetheless, the strong performance of the *in silico* Meta algorithm in our analysis suggests its potential as a valuable aid in prioritizing variants for further investigation, ultimately contributing to more precise genetic diagnoses and personalized medicine.

This study had some limitations that need to be acknowledged. Firstly, due to the exceptional rarity of *KCNK4* variants, only a single case was reported in this study. The phenotypes associated with EFS+ and the entire phenotypic spectrum of *KCNK4* warrant further investigation through large cohort studies. Secondly, the correlation between the spatiotemporal expression of *KCNK4* and its phenotypic features is insufficient to establish a causal link, necessitating additional research to solidify any conclusions. Thirdly, the gain-of-function effects of the variant p.Gly139Arg were only supported by *in silico* analysis, which needed experimental verification.

Our findings suggest that *KCNK4* is a potential novel causative gene for EFS+, which could have important implications for genetic diagnosis and clinical management. The phenotypic spectrum of *KCNK4*-related diseases ranges from mild epilepsy to severe FHEIG syndrome, with moderate EFS+ with partial FHEIG features in between. Further studies are needed to confirm these findings and explore the potential efficacy of sodium channel blockers in patients with EFS + caused by *KCNK4* variants.

## Data Availability

The original contributions presented in the study are publicly available. This data can be found in the GenBank database (accession number: BankIt2932019 PV241702).
